# Antigen Presentation and Autophagy in Teleost Adaptive Immunity

**DOI:** 10.3390/ijms23094899

**Published:** 2022-04-28

**Authors:** Carolina Johnstone, Elena Chaves-Pozo

**Affiliations:** 1 Centro Oceanografico Malaga (COMA-IEO), CSIC, Puerto Pesquero s/n, Fuengirola, 29640 Malaga, Spain; carolina.johnstone@ieo.csic.es; 2 Centro Oceanografico Murcia (COMU-IEO), CSIC, Carretera de la Azohia s/n, Puerto de Mazarron, 30860 Murcia, Spain

**Keywords:** antigen processing, antigen-presenting cell, bacteria, chaperone-mediated autophagy, LC3-Associated phagocytosis, macroautophagy, major histocompatibility complex MHC class I, MHC class II, vaccine, virus

## Abstract

Infectious diseases are a burden for aquaculture. Antigen processing and presentation (APP) to the immune effector cells that fight pathogens is key in the adaptive immune response. At the core of the adaptive immunity that appeared in lower vertebrates during evolution are the variable genes encoding the major histocompatibility complex (MHC). MHC class I molecules mainly present peptides processed in the cytosol by the proteasome and transported to the cell surface of all cells through secretory compartments. Professional antigen-presenting cells (pAPC) also express MHC class II molecules, which normally present peptides processed from exogenous antigens through lysosomal pathways. Autophagy is an intracellular self-degradation process that is conserved in all eukaryotes and is induced by starvation to contribute to cellular homeostasis. Self-digestion during autophagy mainly occurs by the fusion of autophagosomes, which engulf portions of cytosol and fuse with lysosomes (macroautophagy) or assisted by chaperones (chaperone-mediated autophagy, CMA) that deliver proteins to lysosomes. Thus, during self-degradation, antigens can be processed to be presented by the MHC to immune effector cells, thus, linking autophagy to APP. This review is focused on the essential components of the APP that are conserved in teleost fish and the increasing evidence related to the modulation of APP and autophagy during pathogen infection.

## 1. Introduction

### Antigen Processing and Presentation (APP) in Adaptive Immunity

During the early phases of the development of the adaptive immune system, only T lymphocytes, which are capable of recognizing non-self-peptides are selected. This negative selection allows the activation of the effector functions of surveying T lymphocytes, only when their T cell receptor (TCR) specifically interacts with a foreign peptide presented by a major histocompatibility complex (MHC) protein on an antigen-presenting cell (APC). 

Antigen processing and presentation (APP) is the process through which an antigenic protein is processed to peptides, which are loaded and transported to the cell surface on MHC proteins. Autophagy is a self-digestion process that is highly conserved in evolution and involves the degradation of cytoplasmic material delivered to lysosomes by chaperones or by forming double membrane vesicles called autophagosomes [1]. One of the non-canonical functions of autophagy is to participate in APP for the MHC class I and II activation of T lymphocytes [2,3]. 

The sophisticated adaptive immune system allows the recognition and destruction of pathogens and host cells infected by pathogens. A clear picture of the molecular players in APP has been drawn after almost five decades of research [4]. Although most of the research has been centered in mammals, APP in teleost fish has also been assessed through comparative studies [5]. 

In this review, we present an overview of the molecules involved in APP and autophagy in higher vertebrates and review the molecules conserved in teleost fish genomes and the studies that explore their role in APP and autophagy upon infection with viruses, bacteria or parasites and during vaccination. A selection of approximately 600 articles published between 1991 and 2022 retrieved from PubMed^®^ through searches, including the terms “antigen processing” or “antigen presentation” or “autophagy” and “teleost fish” or “zebrafish”, were examined for the purpose of this review.

## 2. Antigen Processing and Presentation and Autophagy in Higher Vertebrates

### 2.1. Antigen Processing and Presentation in Adaptive Immunity

The genes encoding MHC proteins are the most polymorphic genes of the vertebrate genome—a feature that is key for adaptive immunity. The peptides presented on MHC proteins originate from the processing of proteins that are synthesized intracellularly (endogenous antigens) or extracellularly (exogenous antigens). In turn, the proteolytic processing of antigenic proteins to peptides can take place in the cytosol by the proteasome and other cytosolic proteases or by proteases of secretory or lysosomal compartments. In addition, peptides presented on MHC can also be the product of rapidly degraded defective ribosomal products (DRiPs) [6]. 

Chaperones assist in the binding of peptides to the peptide-binding domain (PBD) of MHC proteins. In higher vertebrates, it has been established that MHC class I binds peptides of 8–11 amino acids to specifically interact with the TCR of CD8+ T lymphocytes, whereas MHC class II binds longer peptides of 12–25 amino acids that interact with the TCR of CD4+ T lymphocytes [7]. Analysis of the antigenic peptides bound to a certain MHC protein has allowed establishing motives for different MHC alleles, the prediction of epitopes or peptides with antigenic potential in proteins and the development of preventive treatments against infectious diseases. Immunoinformatic tools for in silico epitope prediction and vaccine design with applications in fish immunology are envisioned [8].

The MHC assembled on the luminal membrane of the endoplasmic reticulum (ER) is transported from the ER to the cell surface of the APC through different pathways depending on the MHC class [4]. Thus, MHC class I molecules are generally transported directly from the ER to the cell surface, while MHC class II molecules are deviated to endosomal compartments [4]. In the MHC class I conventional APP pathway, the antigens are processed by the proteasome, a large catalytic complex with different subunits, some of which are induced by pro-inflammatory cytokines, and therefore different peptides are produced by different proteasome subtypes [9]. Other cytosolic proteases can also participate in the processing of antigens to peptides. 

Cytosolic peptides are transported to the ER lumen through the transporter associated to antigen processing (TAP), which is formed by two molecules, TAP1 and TAP2, both belonging to the ATP-binding cassette transporter family. The loading of MHC class I to form a trimolecular complex with β2-microglobulin (β2m) and the antigenic peptide occurs in the ER, with the assistance of the oxidoreductase ERp57 and chaperones, such as tapasin (also known as TAPBP, for TAP binding protein) and calreticulin [10]. All these molecules form the MHC class I peptide loading complex (PLC). Recently, tapasin-related protein (TAPBPR), a homolog of tapasin, has been reported to be another key component of the MHC class I PLC and is also involved in peptide editing [11]. The antigenic peptide is bound in the PBD formed by highly variable regions of α1 and α2 domains of MHC class I proteins. 

In mammals, it is well-known that antigenic peptides can reach the ER through TAP-independent mechanisms [12] or be the product of antigen proteolysis by multiple proteases [13], including proteases of secretory compartments, such as the ER-associated aminopeptidase [14,15] or the *trans*-Golgi network protease furin [16]. The loading of peptides outside the ER may occur on empty MHC class I travelling through secretory compartments or in endosomal compartments by peptide exchange in recycling MHC class I [17]. 

In addition, CD8 + T lymphocytes can be primed by the MHC class I cross-presentation of peptides processed from exogenous antigens that are acquired through endocytic mechanisms to be transferred to the cytosolic conventional MHC class I APP pathway or to be processed and loaded on MHC class I in secretory or endosomal compartments [18]. Autophagy has been related to TAP- and proteasome-independent MHC class I cross-presentation [19,20].

In the MHC class II APP pathway, the loading of the antigenic peptide occurs in endosomes or lysosomes. The transport of MHC class II molecules to endosomal compartments depends on the association with the chaperone MHC class II invariant chain, li or CD74, an important regulator of immunity and inflammation that binds to the PBD of the MHC protein during the assembly of the nascent MHC class II molecules in the ER [21]. Then li is cleaved by endosomal cysteine proteases, particularly cathepsins S and L [22], leaving only a small fragment termed class II-associated invariant chain peptide (CLIP) protecting the PBD formed by highly variable α1 and β1 domains of MHC class II proteins [23]. The chaperone HLA-DM then assists in peptide editing, in the exchange of CLIP by a high affinity antigenic peptide with an adequate motif to bind to the PBD [24,25]. Autophagy has been shown to play a prominent role in MHC class II APP [26,27,28].

### 2.2. Autophagy in Adaptive Immunity

The substrates of autophagy are widely variable, from mitochondria (“mitophagy), lipid droplets (“lipophagy”), ER (“reticulophagy”), nucleus (“nucleophagy”) or bacteria and viruses (“xenophagy”) [1]. The fact that autophagy operates on cytoplasmic material is the key aspect that differentiates it from other vesicular processes that culminate in the lysosomes and share part of the autophagic machinery, such as phagocytosis or receptor-mediated endocytosis [1].

Chaperone-mediated autophagy (CMA) is the targeting of cytosolic proteins to lysosomes for degradation without requiring vesicle formation and reaching the lysosomal lumen through a protein-translocation complex [29]. A pentapeptide CMA-targeting motif, a KFERQ-like motif, is recognized by a constitutively expressed chaperone, encoded by the gene *hspa8*, the cytosolic protein heat shock cognate protein of 70 KDa (Hsc70), which is the key chaperone in CMA, although other chaperones assist in protein unfolding [29]. In addition, translocation to the lysosomes during CMA requires the binding of the unfolded substrate proteins to monomeric forms of lysosome-associated membrane protein type 2A (Lamp2a), which assemble into a multimeric translocation complex at the lysosomal membrane to release the substrate proteins into the lysosomes [29].

Macroautophagy has been characterized extensively and involves major morphological changes in vesicular compartments to form double-membrane vesicles named as autophagosomes, which engulf large portions of cytoplasm and then fuse with lysosomes [1]. Starvation conditions and stress induce the inactivation of the mammalian target of rapamycin (mTOR) and initiation of macroautophagy. Several multiprotein complexes including a subset of autophagy-related (ATG) proteins are required for the formation of an isolation membrane or phagophore: the unc-51-like kinase (ULK) complex (including proteins ULK1, ATG13, FIP200 and ATG101) and the class III phosphatidylinositol 3-kinase (PI3K) complex (including proteins beclin 1 (BECN1) and VPS34), which increases the local concentration of phosphatidylinositol 3-phosphate marking membranes for autophagosome formation [30]. 

The phagophore expansion to engulf cytoplasmic material and closure in autophagosomes requires the participation of WIPI2 and enzymes ATG7 and ATG10 in the formation of a third multiprotein complex, the ATG16L1 complex (comprising ATG16L1, ATG5 and ATG12), a ubiquitin-like conjugation system, which facilitates the lipidation of microtubule-associated protein 1 light chain 3 (MAP1LC3, also named LC3), which is a homologue of yeast ATG8 or γ aminobutyric receptor-associated protein (GABARAP) [30,31]. The lipidation of LC3-I to formed LC3-II is frequently used as a marker of autophagosome formation [31]. Among the key genes to assess in the study of inhibition or induction of autophagy are *BECN1*, *ATG7*, *LC3*/*GABARAP* and *ULK1* [31].

A closely related process to autophagy, but not a strict form of autophagy, is LC3-associated phagocytosis (LAP). During LAP, LC3 is recruited to a phagosome, which requires some of the core proteins in autophagy, such as ATG5 or ATG7, but does not involve other key autophagy players, such as ULK1 [30]. LAP is not a form of autophagy because phagosomes engulf extracellular material that never reaches the cytosol [30]. Importantly, the use of LC3 as an exclusive marker of autophagy must therefore be interpreted with caution.

## 3. Antigen Processing and Presentation in Teleost Fish

**Figure 1:**
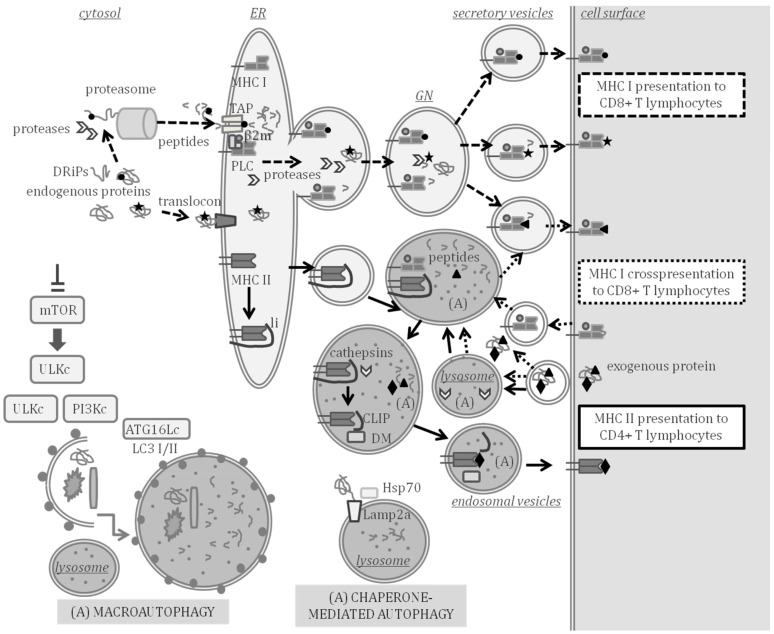
Major histocompatibility complex (MHC) class I and II antigen processing and presentation (APP) and autophagy. MHC class I presents endogenous peptides produced in the cytosol (peptide represented by a circle) or in secretory compartments (star) to CD8 + T lymphocytes through the pathways indicated with the dashed arrows. Endogenously synthesized proteins and defective ribosomal products (DRiPs) are processed to peptides in the cytosol by the proteasome and cytosolic proteases. Peptides are then transported by transporter associated to antigen processing (TAP) to the ER where the peptide loading complex (PLC) is formed by a peptide, TAP, MHC class I, β2-microglobulin (β2m) and other assistant proteins. Endogenous proteins can enter the lumen of the ER through the translocon and be processed by proteases of the ER or the Golgi network (GN), thus, producing peptides that can also be loaded on MHC class I beyond the ER. Loaded MHC class I molecules are transported to the cell surface in secretory vesicles. MHC class I can also cross present peptides and prime CD8+ T lymphocytes through the pathway indicated with the dotted arrows. Cross-presented peptides (triangle) are produced by processing exogenous antigens in the cytosol or in secretory or endocytic compartments in which autophagy (A) may take place. MHC class II presents peptides (diamond) processed from exogenous proteins to CD4 + T lymphocytes through the pathway indicated with the continuous lined arrows. In the ER, MHC class II molecules are assembled and bind to the invariant chain li. The MHC class II is exported to endosomal compartments, in which autophagy can take place and where class II-associated invariant chain peptide (CLIP) remains bound to MHC class II after cleavage of li by lysosomal cathepsins. HLA-DM assists in the exchange of CLIP by a peptide that is exported to the cell surface through exocytic vesicles. Chaperone-mediated autophagy allows the targeting of cytosolic proteins to be degraded in lysosomes and is assisted by Lamp2a, Hsp70 and other chaperones. Macroautophagy is the engulfment of diverse cytosolic material to be degraded, induced by inhibition of the mammalian target of rapamycin (mTOR). Several multiprotein complexes (ULK, PI3K and ATG16L1) are involved in the formation of vesicles and lipidation of LC3-I to LC3-II, thereby, forming autophagosomes that fuse with lysosomes.

The demonstration of APP occurrence in teleost fish came from studies with the channel catfish (*Ictalurus punctatus*) with evidence indicating that MHC-like molecules govern APP with a similar restriction as observed in mammals [32] and with exogenous antigens being endocytosed by APC and inducing the proliferation of lymphocytes [33]. There is sufficient evidence supporting that the functions of MHC class I and II molecules in fish and mammals are similar [34]. A scheme of mammal APP indicating the key molecular players that have been studied and characterized in teleost fish is shown in Figure 1, indicating the compartments where APP and autophagy can take place at the same time.

### 3.1. Antigen Processing and Presentation Genes Conserved in Teleost Fish

The emergence of adaptive immunity is related to the highly polymorphic *mhc* genes encoding MHC, which in most jawed vertebrates are linked, scattered throughout a chromosomal genomic region with varying levels of recombination that also encodes other genes related to APP. Teleost fish are, however, the exception, as the *mhc* class I gene family is expanded and located on several chromosomes, and the *mhc* class II genes are lost in some fish species [35,36]. In spite of the loss of key components of the adaptive machinery, teleost fish are capable of mounting a robust immune response to pathogens [5]. 

Interestingly, the polymorphism in *mhc* class I observed in many teleosts is proposed to be driven by additional evolutionary pressures apart from pathogen resistance [34]. Initial studies in comparative genomics with medaka (*Oryzias latipes*) revealed the evolutionarily conserved core in teleost APP is *mhc* class I and its related genes [37] with the genes encoding MHC class I, proteasome subunits LMP2, LMP7 and TAP2 appearing as linked in linkage analysis [37]. Genes related to MHC class I APP that have been cloned and characterized in teleost fish include several proteasome subunits [38,39,40], *β2m* [41] or the ERp57 gene *pdia3* [42,43] and the tapasin gene *tapbp* [44]. 

The *mhc* class I genes are organized in teleost fish in *U*, *Z*, *L*, *S* or *P* lineages, although not all species have all lineages. Only the *U* lineage is predicted to encode the genes that provide APP functions, displaying haplotypic variation and sharing conserved synteny with the core *MHC* that is observed in mammalian genomes [45]. The *mhc* class I genes have been cloned and characterized in a wide number of teleost fish [41,46,47,48]. The zebrafish (*Danio rerio*) shows substantial diversity within genes of proteasome subunits, *tap* and *mhc* class I genes [49], indicating teleosts have polymorphisms within the MHC class I APP steps of cleavage, transport and presentation, all predicted to alter peptide specificity. 

Vertebrate genome duplication events in evolution have provided cyprinids, salmonids and neoteleosts with duplicates of genes involved in MHC class I APP and therefore with the potential of different PLC [50]. The *U* lineage in the zebrafish is located on chromosome 19, although two *U* genes map to chromosome 22 and are predicted to play non classical roles in APP because they lack key residues for peptide binding and display limited polymorphic variation [51]. Further studies are needed to decipher the complexity in the number of functional genes involved in MHC class I APP, as gene duplicates respond differently to stimulation and may not share biological functions [52].

The *mhc* class II genes in teleost fish are classified in three major groups (*DA*, *DB* and *DE*), with only *DA* exhibiting the classical features [45]. The gene coding for the α chain of class II (*spau-daa*) has been characterized in the gilthead seabream (*Sparus aurata*), showing conserved important features and a high variability in the α1 domain that forms the PBD [53]. The *mhc* class II loci have been lost independently in gadiform fishes, such as Atlantic cod (*Gadus morhua*) [54] and in some syngnathiform fishes, such as the pipefish (*Syngnathus typhle*) [55].

HLA-DM, which assists MHC class II in peptide loading, is highly conserved in tetrapod species. The *HLA-DM*-lineage had only been reported to the level of amphibians until it was recently reported to exist in lungfish [56]. A critical Trp residue required for the HLA-DM system found in mammals is not encoded in classical teleost *mhc* class II and only found in non-classical *mhc* class II genes [57]. The invariant chain (li) and cathepsins, which are involved in the cleavage of li have been studied in depth [58,59,60,61,62,63,64,65,66]. Conserved motifs in the CLIP region of li exist throughout jawed vertebrates. Conserved li residues and cathepsin orthologs suggest their long co-evolution in the APP, with sub-functional duplicated *cd74* genes [56,67]. In rainbow trout (*Oncorhynchus. mykiss*), two *cd74* genes have been characterized, and their expression is regulated differently [58,59].

### 3.2. Antigen-Presenting Cells (APC) and Professional APC (pAPC) in Teleost Fish

All cells are APC as they express MHC class I and can perform APP. Indeed, MHC class I molecules was reported to be expressed in teleost fish in similar cell types as in higher vertebrates [68], and there is evidence indicating that the activation of CD8+ T lymphocyte-mediated cytotoxicity is MHC class I restricted [34,69]. MHC class II is, however, only expressed in the professional APC (pAPC), which are in charge of the initial activation or priming of effector T lymphocytes. 

These pAPC mainly reside in lymphoid organs, such as the head-kidney, the thymus or the spleen, and are capable of priming T lymphocytes to become cytotoxic CD8+ T lymphocytes or CD4+ T helper lymphocytes. The role of pAPC is thus to act as immune sentries that engulf pathogens and carry foreign antigens to lymphoid tissues in order to stimulate an antigen-specific T lymphocyte response. As described in higher vertebrates, the pAPC in some teleost fish species are dendritic cells (DCs), B cells and macrophages, all of which have been characterized in depth in the zebrafish [70]. However, in other species of bony fishes, in addition to DCs, B cells and macrophages, granulocytes [53] and erythrocytes (red blood cells, RBC) [71,72,73] also have a prominent role in APP.

The phenotypic and functional features of DCs have been studied in several teleost species, and methods to enrich DCs have been established. In zebrafish, a pAPC subset strongly resembling mammalian DCs was characterized in relation to affinity for the lectin peanut agglutinin (PNA) [74]. Myeloid cells that were PNA(hi) had phagocytic activity and expressed genes associated to DC function and APP, including MHC class II [74]. Most importantly PNA(hi) cells could activate T lymphocytes in an antigen-dependent manner [74]. 

Among the phenotypic cell surface markers of zebrafish DCs are MHC class II, CD80/86, CD83 and CD209, and all these molecules were involved in the activation of CD4 + T lymphocytes [75]. In barramundi (*Lates calcarifer*), maturation of spleen- and pronephros-derived monocytes exposed in vitro to bacterial-derived peptidoglycans induced differentiation to DC. These DC were phagocytic and could migrate towards pathogens or pathogen associated patterns, they expressed MHC class II and DC-SCRIPT, a conserved zinc finger protein preferentially expressed in human DC subtypes [76]. Importantly, barramundi DCs induced proliferation of effector T lymphocytes [76]. 

In rainbow trout, mucosal tissues from skin and gills have a DC subpopulation that co-expressed CD8α and MHC class II [77], with a homologous population described in intestinal lamina propia [78]. However, the intestinal CD8+ DCs exhibited phenotypic and functional differences compared to skin and gill CD8+ DCs, suggesting the presence of different subpopulations of DCs on different locations [78]. Furthermore, two phenotypes of pAPC were characterized in the gills of rainbow trout. One had features of a monocyte/macrophage/dendritic cell-type; expressing CD45, CD83 and IL-1 β, while the second phenotype expressed almost all genes related to the phagosome, lysosome and APP pathways and exhibited similar characteristics to mammalian M cells, which play a role in mucosal immune surveillance [79]. 

In a different species, the orange-spotted grouper (*Epinephelus coioides*), a transcriptomic study performed on skin after *Cryptocaryon irritans* infection, revealed upregulation of DCs markers, including CD209 and CD83 [80]. CD207 is a marker of Langerhans cells (tissue-resident macrophages of the skin), which are developmentally similar to DCs. Infection of the grass carp (*Ctenopharyngodon idella*) with *Flavobacterium columnare* upregulated CD207 only in the spleen, suggesting that the biological function of CD207 may be related to APP [81].

B cells are pAPC in teleosts as they can uptake particulate antigens by phagocytosis, process and present antigens to T lymphocytes [82,83]. In bony fish, B cells produce three isotypes of immunoglobulins (Ig): IgM, IgD and IgT/Z [82]. In accordance, three main B-cell lineages have been described in teleosts, a B-cell lineage expressing IgT/Z, a lineage expressing IgD and the most common B-cell lineage co-expressing IgD and IgM [82]. 

In the rainbow trout, infection in vitro of blood or spleen IgM+ B cells with viral hemorrhagic septicemia virus (VHSV) upregulates MHC class II, CD80/86 or CD83, suggesting the activation towards a pAPC profile [84]. In vivo IgM+ B cells are recruited to the peritoneum cavity after challenge with virus VHSV or bacteria *Escherichia coli* and differentiate to cells specialized in APP [85]. A teleost B cell subset has been proposed to be a counterpart of mammalian B1-B cells, which link innate and adaptive immunity [86].

Professional phagocytic cells have a role in pAPC in teleost fish. Thus, macrophages are pAPC that participate in immunity as phagocytic cells, with two polarization states, the pro-inflammatory M1-type and anti-inflammatory M2-type. The expression of genes involved in APP was detected in both M1- and M2-type macrophages in the zebrafish [87]. In addition, the infection of macrophages upregulated MHC class II and transcripts of other genes involved in APP [87]. In addition to macrophages, head-kidney granulocytes can express high levels of MHC class II in gilthead seabream [53] or in Atlantic salmon (*Salmo salar*) [71] and are proposed as a model to study autophagy and APP during vertebrate immune response [71].

### 3.3. Peptide Processing, Loading and Transport by MHC in Teleost Fish

A number of studies have characterized some of the key proteins involved in the processing of antigens to peptides, the loading on MHC and MHC/peptide transport to the cell surface of APC. Concerning the MHC class I APP pathway, in European sea bass (*Dicentrarchus labrax*), different transcripts of MHC class I have been identified per individual, detecting variability at the PBD, with a three-dimensional homology model that is consistent with that of other vertebrates [41]. The proteasome activator subunits PA28α and PA28β were upregulated upon immune stimulation in the Japanese flounder (*Paralichthys olivaceus*) [38], the large yellow croaker (*Larimichthys crocea*) [39] and the rock bream (*Oplegnathus fasciatus*) [40]. 

The expression of β2m was induced by an antigen in the European sea bass [41]. The interaction of tapasin with TAP, MHC class I and ERp57, all which are known to occur during assembly of the mammalian peptide loading complex (PLC), was conserved in the monocyte/macrophage rainbow trout cell line RTS11 [88]. Homology models of the protein ERp57 in European sea bass support that they are orthologs of the mammalian protein involved in MHC class I APP [42]. 

In rainbow trout, expression analysis supports the role of ERp57 during the activation of the immune response in teleosts [43], although some features conserved in mammalian ERp57 are not conserved in the fish gene [43]. In the same species, tapasin was mainly expressed in immune system organs and in cell lines derived from epithelial cells from the gill, liver and intestine [44]. In addition, tapasin was strongly expressed in parallel with MHC class I in the RTS11 monocyte/macrophage derived cell line after viral infection [44].

Concerning the MHC class II APP pathway, the expression of the alpha chain of gilthead seabream MHC class II (*Spau-DAA*) was observed in peritoneal exudate leucocytes, head-kidney, spleen, thymus and gill; and the incubation of head-kidney leucocytes with yeast or bacteria upregulated levels of *Spau-DAA*, whereas concanavalin A (ConA), lipopolysaccharide (LPS) or phytohemagglutinin A (PHA) did not [53]. 

In the MHC class II APP, the interferon-γ-inducible lysosomal thiol reductase (GILT) is important as it reduces disulfide bonds in the unfolding of proteins and facilitates their subsequent cleavage by proteases. In zebrafish, purified recombinant GILT was capable of catalyzing the reduction of protein disulfide bonds [89]. GILT was constitutively expressed in several tissues of zebrafish, large yellow croaker and mandarin fish (*Siniperca chuatsi*) and upregulated in the spleen and kidney after stimulation [89,90,91].

The invariant chain li, a key player in the formation of mammalian MHC class II/peptide complex, appears to be expressed independently of immune function in some teleost fish. The invariant chain-like proteins S25-7 and INVX of the rainbow trout were not expressed in the head-kidney, and their expression in spleen and gill was unaffected by immune stimulation [59]. Cathepsins, the family of lysosomal cysteine proteases that are involved in the processing of li, have been characterized in depth in teleost species. 

Several cathepsins have been characterized and found to be widely expressed and produced in vitro to confirm their protease activity: cathepsins D and L in the half-smooth tongue sole (*Cynoglossus semilaevis*) [61]; in the large yellow croaker cathepsin L and S [62] and B [60]; in the miiuy croaker (*Miichthys miiuy*) cathepsin S [63] and cathepsin B and cathepsin H [64] were assessed; while cathepsin B was studied in the golden pompano (*Trachinotus ovatus*) [66] and cathepsin S in the channel catfish [65]. The capacity of cathepsins to cleave the invariant chain li was studied in the large yellow croaker, finding that cathepsin B efficiently cleaves recombinant li in a time-dependent manner [60]. 

Cathepsin S and cathepsin L both had cysteine protease activity and could remove propeptides and release active mature peptides; however, li inhibited the autocatalytic activation of cathepsin L, whereas activated cathepsin S could efficiently process li in vitro [62]. These results suggest that cathepsins S and B are the main cathepsins involved in the processing of li in teleost fish [60,62]. It should be noted that, in the channel catfish, two cathepsin S genes have been characterized, (*ctss* and *ctssa*), both upregulated during bacterial infection, however, with differences between their expression profiles in mucosal surfaces, suggesting that they may exert disparate roles in mucosal immunity [65].

## 4. Autophagy and Related LC3-Associated Phagocytosis in Teleost Fish

Autophagy is a self-digestion process that is highly conserved in evolution and involves lysosomal degradation of cytoplasmic material [1]. Autophagy mainly occurs by fusion of autophagosomes, which engulf portions of cytosol and fuse with lysosomes (macroautophagy), or assisted by chaperones (chaperone-mediated autophagy, CMA) that deliver cytosolic proteins to lysosomes. One of the non-canonical functions of autophagy is to participate in APP for MHC class I and II activation of T lymphocytes [3]. LC3-associated phagocytosis (LAP), however, is not a form of autophagy; it depends on key autophagy molecular players [30].

### 4.1. Chaperone-Mediated Autophagy in Teleost Fish

CMA was long believed to only exist in birds and mammals [92]; however, it has been proved to exist also in fish [93,94]. Lamp2a is the product of one of the three splice variants (*lamp2a*, *lamp2b* and *lamp2c*) of gene *lamp2* that is transcribed by alternative splicing, and this genomic organization has been highly conserved during vertebrate evolution [93]. The gene *lamp2* appears at the root of the vertebrate lineage with a Ct domain GYXXF sequence that is conserved in most teleosts [93], including medaka. However, *lamp2* is divergent in zebrafish, which raised the question on the ability of this species to perform CMA [92]. 

In medaka in vitro studies, the splice variant *lamp2a* was found to control lysosomal accumulation of a reporter of CMA in a fibroblast cell line; and in vivo, a *lamp2a* medaka knock-out exhibited carbohydrate and fat metabolism alterations [93]. In cultured cells of the yellowtail fish (*Seriola quinqueradiata*), three cytosolic members of the Hsp70 family have been isolated; heat-shock cognate proteins (Hsc)70-1 and Hsc70-2 and heat shock protein (Hsp)70 [95]. Heat-induced expression of Hsp70, together with the lysosome localization of Hsc70/Hsp70 suggests that CMA is induced by heat shock in bony fish [95]. In the zebrafish, a member of the small heat shock protein (sHSP) family, Hspb8, interacts with proteins Bag3 and Hsc70, which are essential for the formation of an autophagy-inducing complex [96].

### 4.2. Macroautophagy in Teleost Fish

Xenophagy is the capture and destruction of invading pathogens by means of a form of selective macroautophagy that requires the participation of receptors Sqstm1/p62 that bind to ubiquitinated cargo and LC3-II to form p62-droplets that accumulate at the surface of autophagosomes during their formation [1,30,31]. The contribution of zebrafish models to study the specific elimination of certain intracellular substrates by autophagic pathways, has been recently reviewed [97], as well as the role of selective autophagy in immunity to bacterial pathogens (*Shigella flexneri* and *Mycobacterium marinum*) [98]. 

Inflammatory toxicity in zebrafish induces the cleavage of p62 by caspase-6 at a highly conserved D256 cleavage site, indicating certain stress stimuli can modulate autophagy through the caspase 6-p62 axis [99]. Zebrafish mutant lines have shown that *becn1* is essential for autophagy, as knock-out mutants for this gene die at the larval stage [100]. In addition, mutants in *becn1* and *atg7* have outlined the importance of *becn1* for maintaining homeostasis of nutrient metabolism and in liver development [101].

### 4.3. LC3-Associated Phagocytosis in Teleost Fish

In a zebrafish embryo model, the interaction of human pathogen *Salmonella typhimurium* with the autophagy machinery of macrophages identified LAP as the major host protective autophagy-related pathway responsible for macrophage defense during systemic infection [102]. In contrast, in an infection model to study the human pathogen *Staphylococcus aureus*, LAP was found to be involved in the intracellular handling of the pathogen by neutrophils [103] with a key role for Sqstm1/p62 in autophagic control of infection [104].

## 5. Regulation of Antigen Processing and Presentation and Autophagy during Infection of Teleost Fish

The regulation of expression of genes of the APP MHC class I and II pathways and of autophagy has been studied in a wide range of organs and cells during the infection of teleost fish by viruses (Table 1) by bacteria and eukaryote parasites (Table 2) or during vaccination (Table 3). It should be stressed that very few studies have assessed APP and autophagy simultaneously.

### 5.1. Modulation of Antigen Processing and Presentation and Autophagy during Viral Infection

Antiviral adaptive immune responses against the viral hemorrhagic septicemia virus (VHSV), a rhabdovirus that causes important economic losses to the aquaculture industry, have been studied in depth in several fish species and types of organs or cells (Table 1).

**Table 1:** Modulation of expression of genes involved in antigen processing and presentation (APP) MHC class I and II pathways and autophagy during infection of teleost fish species with viral pathogens.

Virus ^1^	Host Species	MHC I ^2^ APP	MHC II ^2^ APP	Autophagy ^2^	Organ or Cell	Reference
VHSV	*Lateolabrax japonicus*	n.d.	−	n.d.	Brain cell line.	[105]
	*Sander vitreus*	+	n.d.	n.d.	Skin fibroblast cell line.	[106]
	*Oncorhynchus mykiss*	+	n.d.	n.d.	Skin fibroblast cell line.	[107]
		+	+	+	Red blood cells and head-kidney.	[108,109]
		n.d.	+	n.d.	Blood and spleen B cells.	[84]
	*Scophthalmus maximus*	n.d.	n.d.	+	Red blood cells.	[73]
	*Paralichthys olivaceus*	n.d.	n.d.	+	Kidney and spleen.	[110]
NNV	*Epinephelus coioides*	+	n.d.	n.d.	Liver and spleen.	[47]
	*Sparus aurata*	+	−	−/+	Larvae.	[111]
CSV	*Oncorhynchus mykiss*	+	n.d.	n.d.	Monocyte/macrophage cell line.	[44]
GCRV	*Ctenopharyngodon idella*	n.d.	n.d.	+	Kidney cell line.	[112]
SGIV	*Epinephelus coioides*	+	n.d.	n.d.	Liver and spleen.	[47]
IHNV	*Oncorhynchus mykiss*	+	−	n.d.	Spleen, intestines and head-kidney.	[113]
RBIV	*Cynoglossus semilaevis*	n.d.	+	n.d.	Kidney and spleen.	[61]
SAV3	*Salmo salar*	+	n.d.	n.d.	Pancreas, heart, spleen, head-kidney, liver and gills.	[114]
ISKNV	*Paralichthys olivaceus*	+	n.d.	n.d.	Head-kidney, spleen and liver.	[115]

^1^ Viral pathogens: Chum Salmon Reovirus (CSV), Grass carp reovirus (GCRV), Infectious hematopoietic necrosis virus (IHNV), Infectious spleen and kidney necrosis virus (ISKNV), Nervous Necrosis Virus (NNV), rock bream iridovirus (RBIV), RNA virus salmonid alpha virus 3 (SAV3), Singapore grouper iridovirus (SGIV), Viral Hemorrhagic Septicemia Virus (VHSV); ^2^ Expression of genes related to APP MHC class I pathways, APP MHC class II pathways or autophagy were (+) upregulated, (−) downregulated or (n.d.) not determined.

Upregulation of MHC class I and II APP and also of autophagy genes were observed after VHSV infection in rainbow trout RBCs [108,109]. Interestingly, suboptimal temperatures delayed the enhanced expression of MHC class I APP genes induced by VHSV infection in fibroblast cell lines derived from the rainbow trout [107] or the walleye (*Sander vitreus*) [106]. MHC class II upregulated through IFNγ overexpression was inhibited in a cell line of sea perch (*Lateolabrax japonicus*) after infection with VHSV [105]. In fact, overexpression of the viral N protein promoted proteasomal degradation of IFNγ signaling proteins through enhanced ubiquitination [105], demonstrating viral evasion in teleost fish adaptive immunity.

The upregulation of APP genes or autophagy has also been reported during infection with other viruses, as observed with VHSV. In relation to the MHC class I APP pathway, in the orange-spotted grouper, the expression of MHC class I genotype EcMHC-I A*01, was induced by infection with red-grouper nervous necrosis virus (RGNNV) or Singapore grouper iridovirus (SGIV) in liver and spleen tissues and, in turn, overexpression of EcMHC-I A*01 reduced expression of viral genes [47]. In the Japanese flounder expression and function of MHC class I α homologue *PoMHC Iα* was upregulated after infection with infectious spleen and kidney necrosis virus (ISKNV) [115]. 

In the rainbow trout RTS11 cell line, *tapbp*, a MHC class I APP gene, was strongly expressed in parallel with *mhc* class I after infection with the Chum salmon reovirus (CSV) [44]. In Atlantic salmon, six genes of the *mhc* class I *L* lineage (*Sasa-lia*, *Sasa-lda*, *Sasa-lca*, *Sasa-lga*, *Sasa-lha* and *Sasa-lfa*) were found to be differently upregulated during infection with salmonid alpha virus 3 (SAV3) [114]. 

These findings outline the need for further studies that assess different *mhc* class I lineages in teleost fish, as investigating single *mhc* class I genes may lead to erroneous conclusions. The effect of the viral mimic Poly I:C has also been studied in several teleost species. Upregulation of APP MHC class I genes was observed in a rainbow trout (*O. mykiss*) cell line [44] and in the European sea bass (*Dicentrarchus labrax*) [41], whereas in the Antarctic bullhead notothen (*Notothenia coriiceps*), APP genes were downregulated [116]. 

During acute infection of the rainbow trout (*O. mykiss*) with infectious hematopoietic necrosis virus (IHNV), *mhc* class I and *β2m* were induced whereas *mhc* class II was downregulated [113]. In rainbow trout, VHSV infection in vitro upregulated *mhc* class II [84]. In the case of key players of the MHC class II APP pathway, in the half-smooth tongue sole, cathepsins were induced in the kidney and the spleen after a challenge with rock bream iridovirus (RBIV) [61].

Concerning autophagy, it can be downregulated or impaired during viral infection of teleost fish. Induction of autophagy during VHSV infection appears to be host-beneficial. In the Japanese flounder, the infection induced autophagy through expression of *becn1*, which reduced transcripts of viral glycoprotein G when overexpressed [110]. In accordance, blocking autophagy in VHSV-infected cells of the flatfish turbot resulted in increased viral replication [73]. Therefore, there is sufficient evidence pointing to the interest of investigating strategies that upregulate APP and autophagy as rhabdovirus preventive treatments. 

The antiviral activity of the zebrafish C-reactive protein-like isoform CRP1-7 against spring viremia of carp virus (SVCV) was related to inhibition of autophagy, by disturbing cholesterol ratios in the host cellular membranes, which negatively affected the intracellular regulation of reactive oxygen species (ROS) and consequently increased lysosomal pH [117]. In zebrafish mutants in the tumor suppressor phosphatase and tensin homolog deleted on chromosome 10 genes *ptena* and *ptenb*, infection with SVCV was slower and may be related to impaired autophagy [118]. 

Concerning CMA, the multifunctional cellular protein high-mobility group box 1b (Hmgb1b) acts as a heat shock protein 70 (Hsp70)-dependent, pro-autophagic protein in grass carp during infection with grass carp reovirus (GCRV) [112]. Finally, downregulation of autophagy was reported in a transcriptomic study in the gilthead seabream during early infection with nervous necrosis virus genotype RGNNV/SJNNV, at the same time heat shock protein transcripts (including Hsc70) and MHC class I APP genes were upregulated [111]. Interestingly, at late times after infection, autophagy was upregulated and MHC class II APP genes downregulated [111].

### 5.2. Modulation of Antigen Processing and Presentation and Autophagy during Bacterial or Parasite Infection

As reported for viruses, infection of teleost fish with pathogenic bacteria generally upregulates APP and autophagy and is mainly host-beneficial (Table 2). Concerning MHC class I APP, in Atlantic salmon *Piscirickettsia salmonis* can upregulate the expression of a *mhc* class I gene [114]. The expression of *PoMHCIα* in the Japanese flounder (*Paralichthys olivaceus*) was upregulated after infection with *E. tarda* and *V. anguillarum* [115]. A microarray analysis of the expression of genes in liver of the blue catfish (*Ictalurus furcatus*) after infection with *Edwardsiella ictaluri* showed a strong upregulation of several pathways including APP with induction of MHC class I related genes [119]. 

**Table 2:** Modulation of expression of genes involved in antigen processing and presentation (APP) MHC class I and II pathways and autophagy during infection of teleost fish species by bacteria or eukaryote parasites.

Pathogen ^1^	Host Species	MHC I ^2^ APP	MHC II ^2^ APP	Autophagy ^2^	Organ or Cell	Reference
*Piscirickettsia salmonis*	*Salmo salar*	+/(=)	n.d.	n.d.	Heart, spleen, head-kidney, liver and gill.	[114]
*Vibrio anguillarum*	*Sebastes schlegelii*	+	n.d.	−	Lymphocytes from peripheral blood, head-kidney and spleen.	[120]
	*Sparus aurata*	n.d.	+	n.d.	Head-kidney leucocytes.	[53]
	*Cynoglossus semilaevis*	n.d.	+	n.d.	Kidney and liver.	[61]
	*Miichthys miiuy*	n.d.	+	n.d.	Liver, spleen and kidney.	[64]
	*Paralichthys olivaceus*	+	n.d.	n.d.	Head-kidney, spleen and liver.	[115]
*Yersinia ruckeri*	*Oncorhynchus mykiss*	n.d.	+	n.d.	Head-kidney and spleen.	[121]
*Edwarsiella ictaluri*	*Ictalurus furcatus*	+	n.d.	n.d.	Liver.	[119]
	*Ictalurus punctatus*	n.d.	+	n.d.	Skin, gill and intestine.	[65]
*Edwardsiella tarda*	*Paralichthys olivaceus*	+	n.d.	n.d.	Head-kidney, spleen and liver.	[115]
	*Trachinotus ovatus*	n.d.	+	n.d.	Liver, spleen and head-kidney.	[66]
*Citrobacter freundii*	*Danio rerio*	+	−	+	Skin.	[122]
*Streptococcus agalactiae* ^3^	*Danio rerio*	−	−	n.d.	Intestine and skin.	[123]
*Staphylococcus aureus* ^3^	*Danio rerio*	n.d.	n.d.	+	Macrophages and neutrophils.	[98,103]
*Salmonella typhimurium* ^3^	*Danio rerio*	n.d.	n.d.	+	Macrophages and neutrophils.	[98]
		n.d.	n.d.	+/−	Macrophages and larvae.	[102,124]
*Saccharomyces cerevisiae*	*Sparus aurata*	n.d.	+	n.d.	Head-kidney leucocytes.	[53]
*Enteromyxum scophthalmi*	*Scophthalmus maximus*	−	n.d.	n.d.	Blood.	[72]
*Neoparamoeba perurans*	*Salmo salar*	−	n.d.	n.d.	Gills.	[125]
*Lepeophtheirus salmonis*	*Salmo salar*	−	−	n.d.	Skin and head-kidney.	[126]
*Gyrodactylus bullatarudis*	*Poecilia reticulata*	+	=	n.d.	Skin.	[127]
*Cryptocaryon irritans*	*Epinephelus coioides*	+	+	n.d.	Skin.	[80]
	*Sparus aurata*	n.d.	−/=	n.d.	Gills and head-kidney.	[128]

^1^ Prokaryote bacteria or eukaryote parasites separated by line; ^2^ Expression of genes related to APP MHC class I pathways, APP MHC class II pathways or autophagy were (+) upregulated, (−) downregulated, (=) not modulated or (n.d.) not determined. ^3^ Human pathogen studied in zebrafish infection model.

In relation to MHC class II APP, the expression of *Spau-DAA* was upregulated by bacterial infection of gilthead seabream head-kidney leucocytes [53]. Fructose 1,6-biphosphate aldolase (FBA), a conserved enzyme of the glycolytic pathway, provides protection against various aquaculture pathogenic bacteria, and this could be related to the increased expression of *mhc* class II observed in treated zebrafish and turbot [129]. The induction of cathepsins has also been reported in several fish species during bacterial infection. Cathepsins D and L are induced in kidney and spleen during infection with *Vibrio anguilarum* in the half-smooth tongue sole [61], and in the miiuy croaker, cathepsins B and H were upregulated in the liver, spleen and kidney [64]. 

In golden pompano, cathepsin B is overexpressed after infection with bacteria *Edwardsiella tarda* [66]. In rainbow trout, genes found to be differentially expressed in head-kidney and spleen upon infection by *Yersinia ruckeri* included cathepsin B and lysosomal genes [121]. In channel catfish, the two cathepsin S genes that have been characterized were upregulated during infection with *Edwardsiella ictaluri* [65]. Certain studies have assessed regulation of APP through bacterial infection using bacterial antigens as stimuli. 

In yellowtail fish (*Seriola quinqueradiata*) a microarray study after stimulation with ConA or LPS of primary cultured kidney cells resulted in induced expression of genes related to APP including MHC class II β chain [130]. In contrast to the effect of Poly I:C in the Antarctic bullhead notothen, exposure to a bacterial ligand (heat-killed *Escherichia coli*) induced both MHC class I and class II APP pathway genes [116]. In contrast, both MHC class I and II APP genes were downregulated in intestines and skin by bacterial infection in a zebrafish infection model with *Streptococcus agalactiae* [123].

The zebrafish is an infection model for the study of human bacterial pathogens, sometimes related to fish consumption. Those studies have also contributed to decipher the host protective or detrimental role of autophagy and LAP. A larvae infection model with *Shigella flexneri* indicated that intracellular engulfed bacteria can escape to the cytosol and be targeted to autophagy [131]. In fact, depletion of SQSTM1/p62 resulted in an increase in host susceptibility to infection and bacterial burden [131]. 

A zebrafish model to study tuberculosis, caused by *Mycobacterium tuberculosis*, was developed by infecting fish with the closely related *Mycobacterium marinum*, unveiling the function of DNA-damage regulated autophagy modulator 1 (DRAM1) gene *dram1* in autophagy. DRAM1 had a host-protective effect promoting the formation of autophagosomes in a mechanism that depended on SQSTM1/p62 [132,133]. DRAM1 has also been linked to the antimicrobial response in a *Salmonella* infection model [134]. 

In rockfish (*Sebastes schlegelii*), infection with *V. anguillarum* increased expression of Raptor and cytotoxicity related genes, and inhibiting mTORC1 signaling through rapamycin, a classic activator of autophagy, led to impaired lymphocyte-mediated cytotoxic responses making fish more vulnerable to infection by bacteria [120]. In the model of infection with *Salmonella Typhimurium*, which contains a *spvB* gene encoding a protein related to higher virulence, the *spvB* gene increased bacterial survival and intestine injuries due to inhibition of autophagy [124]. 

Regarding LAP, it can be both beneficial for the host—in the case of infection with *Salmonella typhimurium*—but detrimental in the case of infection by *Staphylococcus aureus*, which is delivered to a replication niche by LAP. [98]. Simultaneous assessment of APP and autophagy has been scarcely investigated. A study on the gene expression profile of the zebrafish skin upon bacterium *Citrobacter freundii* infection, showed that MHC class I APP and autophagy related genes were upregulated, whereas MHC class II APP was downregulated [122].

Finally, several studies have assessed modulation of APP in parasitized fish (Table 2). The expression of *Spau-DAA* was upregulated by yeast infection of gilthead seabream head-kidney leucocytes [53]. In Atlantic salmon, downregulation of MHC class I APP genes was also reported in relation to the amoebic gill disease [125] and to the ectoparasitic copepod salmon louse (*Lepeophtheirus salmonis*) that also downregulated MHC class II in skin [126]. 

The immune primary response to the ectoparasite *Gyrodactylus bullatarudis* has been characterized in the skin of guppies (*Poecilia reticulata*), finding MHC class I APP genes were upregulated, whereas MHC class II was not significantly increased [127]. In the Orange-spotted grouper (*Epinephelus coioides*) skin infection with ectoparasite *Cryptocaryon irritans* upregulated expression of MHC class I and II APP genes [80]. However, the same parasite downregulated the expression of MHC II alpha chain in the head-kidney of gilthead seabream specimens but not in gills—one of the target tissues of the ectoparasite [128]. Infection by the myxozoan parasite *Entermyxum scophthalmi* has been studied through a blood transcriptional profile in turbot (*Scophthalmus maximus*), reporting downregulation of APP after infection [72].

### 5.3. Modulation of Antigen Processing and Presentation and Autophagy upon Vaccination

Vaccination of teleost fish to fight viral and bacterial diseases induces APP pathways or modulates autophagy (Table 3). Concerning APP pathways, the *Edwardsiella tarda* live attenuated vaccine “WED” remarkably affected APP genes in zebrafish as shown by Gene Ontology and Kyoto Encyclopedia of Genes and Genomes database analysis in a transcriptomic approach [135]. 

**Table 3:** Modulation of expression of genes involved in antigen processing and presentation (APP) MHC class I and II pathways and autophagy during vaccination of teleost fish species.

Vaccine ^1^	Host Species	MHC I ^2^ APP	MHC II ^2^ APP	Autophagy ^2^	Organ or Cell	Reference
*Vibrio harvey*	*Cynoglossus semilaevis*	n.d.	+	n.d.	Kidney.	[61]
*Edwardsiella tarda*	*Danio rerio*	+	−	n.d.	Liver and spleen.	[135,136]
*Vibrio anguillarum and Edwardsiella tarda*	*Scophthalmus maximus*	+	+	n.d.	Liver and spleen, kidney.	[137]
	*Danio rerio*	+	+	n.d.	Liver and spleen.	[137]
*Vibrio parahaemolyticus, Vibrio alginolyticus and Aeromonas hydrophila*	*Larimichthys crocea*	+	+	n.d.	Spleen.	[138]
VHSV	*Danio rerio*	n.d.	n.d.	+	Fibroblast cell line and muscle.	[139]
SVCV	*Danio rerio*	n.d.	n.d.	+	Fibroblast cell line and muscle.	[139]

^1^ Vaccines addressed to the indicated pathogens. Spring viremia of carp virus (SVCV), Viral Hemorrhagic Septicemia Virus (VHSV); ^2^ Expression of genes related to APP MHC class I pathways, APP MHC class II pathways or autophagy were (+) upregulated, (−) downregulated or (n.d.) not determined.

In different immunization protocols against *E. tarda,* genes related to MHC class I and CTL responses were up-regulated, whereas MHC class II APP genes and CD4+ T lymphocyte responses were down-regulated [135,136], suggesting the mechanism behind the protective immunity conferred was related to induction of MHC class I APP and that CTL function played a major protective role in *E. tarda* infection in the zebrafish [136]. A combinatory vaccination approach consisting of *E. tarda* WED and *V. anguilarum* MVAV6203 vaccines, upregulated APP genes, including *mhc* class I and class II, in the liver and spleen of the zebrafish and of the turbot [137]. 

An inactivated trivalent vaccine (*Vibrio parahaemolyticus*, *Vibrio alginolyticus* and *Aeromonas hydrophila*) has been studied through transcriptomics in the spleen of the large yellow croaker, revealing genes involved in MHC class I and II APP pathways were upregulated [138]. The expression of cathepsins, MHC class II APP related genes, was induced in the half-smooth tongue sole (*Cynoglossus semilaevis*) upon vaccination with the *Vibrio harveyi* subunit vaccine DegQ [61]. In relation to autophagy, vaccination of zebrafish with G glycoproteins of VHSV and SVCV elicited strong antiviral responses during which autophagy was activated [139] and necessary for viral clearance [140].

## 6. Conclusions

MHC class I APP is highly conserved in teleost fish, with the MHC class I *U* lineage predicted to encode the genes that provide APP functions. In contrast to tetrapods, the *mhc* class I gene family in teleost fish has expanded and is located on several chromosomes; however, gene duplicates respond differently to stimulation and may not share biological functions. The key elements of the MHC class I PLC are conserved in teleost fish, and the variability per individual and at the PBD suggests that peptides are selected according to conserved motifs.

Several species of teleost fish have lost MHC class II but not others. In teleost fish that conserve the *mhc* class II genes, the *DA* lineage exhibits the classical MHC class II features. In teleosts, the expression of the invariant chain li is regulated differently to higher vertebrates, with sub-functional duplicated *cd74* genes. Conserved li residues and cathepsin orthologs suggest their long co-evolution in the APP, and cathepsins B and S may be the main cathepsins involved in the processing of li in teleost fish. The chaperone HLA-DM is only reported in some teleost species. In teleost fish, pAPC are DCs, B cells and macrophages; however, in addition, RBC and granulocytes have a prominent role in APP in some bony fish species.

One of the non-canonical functions of autophagy is to participate in APP for MHC class I and II activation of T lymphocytes; however, this aspect of autophagy is barely studied in teleost fish. Most of the literature refers to the contribution of zebrafish models of infection by human pathogens to study LAP, which can be either beneficial or detrimental for the host. Chaperone-mediated autophagy (CMA) exists in fish and deserves further studies to determine its role upon infection.

There are few studies assessing APP and autophagy simultaneously. However, in general, the upregulation of MHC class I and II APP pathways as well as of autophagy is reported in an important number of pathogen–host systems. In certain pathogen–fish host systems, MHC class I and class II APP pathways are modulated differently or autophagy is downregulated or impaired during infection. Interestingly, the scarce vaccines in which APP pathways and autophagy have been studied modulate this cellular process. All this complexity points to the need to display further studies to unravel the role of APP and autophagy in fish disease and pathogen evasion that allow the design of preventive and palliative molecular tools to fight against infection in commercial fish species.

## Data Availability

Not applicable.

## References

[1] Galluzzi L., Baehrecke E.H., Ballabio A., Boya P., Bravo-San Pedro J.M., Cecconi F., Choi A.M., Chu C.T., Codogno P., Colombo M.I. (2017). Molecular definitions of autophagy and related processes. EMBO J..

[2] Münz C. (2021). The macroautophagy machinery in MHC restricted antigen presentation. Front. Immunol..

[3] Münz C. (2021). Non-canonical functions of autophagy proteins in immunity and infection. Mol. Asp. Med..

[4] Rock K.L., Reits E., Neefjes J. (2016). Present yourself! By MHC class I and MHC class II molecules. Trends Immunol..

[5] Wilson A.B. (2017). MHC and adaptive immunity in teleost fishes. Immunogenetics.

[6] Yewdell J.W., Antón L.C., Bennink J.R. (1996). Defective ribosomal products (DRiPs): A major source of antigenic peptides for MHC class I molecules?. J. Immunol..

[7] Wu Y., Zhang N., Hashimoto K., Xia C., Dijkstra J.M. (2021). Structural comparison between MHC classes I and II; in evolution, a Class-II-like molecule probably came first. Front. Immunol..

[8] De Groot A.S., Moise L., Terry F., Gutierrez A.H., Hindocha P., Richard G., Hoft D.F., Ross T.M., Noe A.R., Takahashi Y. (2020). Better epitope discovery, precision immune engineering, and accelerated vaccine design using Immunoinformatics tools. Front. Immunol..

[9] Abi Habib J., Lesenfants J., Vigneron N., Van den Eynde B.J. (2022). Functional differences between proteasome subtypes. Cells.

[10] Blees A., Januliene D., Hofmann T., Koller N., Schmidt C., Trowitzsch S., Moeller A., Tampé R. (2017). Structure of the human MHC-I peptide-loading complex. Nature.

[11] Thomas C., Tampé R. (2021). MHC I assembly and peptide editing—chaperones, clients, and molecular plasticity in immunity. Curr. Opin. Immunol..

[12] Johnstone C., Del Val M. (2007). Traffic of proteins and peptides across membranes for immunosurveillance by CD8+ T lymphocytes: A topological challenge. Traffic.

[13] Del-Val M., López D. (2002). Multiple proteases process viral antigens for presentation by MHC class I molecules to CD8+ T lymphocytes. Mol. Immunol..

[14] Serwold T., Gonzalez F., Kim J., Jacob R., Shastri N. (2002). ERAAP customizes peptides for MHC class I molecules in the endoplasmic reticulum. Nature.

[15] Chang S.-C., Momburg F., Bhutani N., Goldberg A.L. (2005). The ER aminopeptidase, ERAP1, trims precursors to lengths of MHC class I peptides by a “molecular ruler” mechanism. Proc. Natl. Acad. Sci. USA.

[16] Gil-Torregrosa B.C., Castaño A.R., Del Val M. (1998). Major histocompatibility complex class I viral antigen processing in the secretory pathway defined by the trans-Golgi network protease furin. J. Exp. Med..

[17] Grommé M., Uytdehaag F.G.C.M., Janssen H., Calafat J., van Binnendijk R.S., Kenter M.J.H., Tulp A., Verwoerd D., Neefjes J. (1999). Recycling MHC class I molecules and endosomal peptide loading. Proc. Natl. Acad. Sci. USA.

[18] Rock K.L., Shen L. (2005). Cross-presentation: Underlying mechanisms and role in immune surveillance. Immunol. Rev..

[19] Possamaï D., Hanafi L.A., Bellemare-Pelletier A., Hamelin K., Thébault P., Hébert M.J., Gagnon É., Leclerc D., Lapointe R. (2021). MHC class I antigen cross-presentation mediated by PapMV nanoparticles in human antigen-presenting cells is dependent on autophagy. PLoS ONE.

[20] Johnstone C., Ramos M., García-Barreno B., López D., Melero J.A., Del Val M. (2012). Exogenous, TAP-independent lysosomal presentation of a respiratory syncytial virus CTL epitope. Immunol. Cell Biol..

[21] Schröder B. (2016). The multifaceted roles of the invariant chain CD74—More than just a chaperone. Biochim. Biophys. Acta.

[22] Chapman H.A. (1998). Endosomal proteolysis and MHC class II function. Curr. Opin. Immunol..

[23] Romagnoli P., Germain R.N. (1994). The CLIP region of invariant chain plays a critical role in regulating major histocompatibility complex class II folding, transport, and peptide occupancy. J. Exp. Med..

[24] Reyes-Vargas E., Barker A.P., Zhou Z., He X., Jensen P.E. (2020). HLA-DM catalytically enhances peptide dissociation by sensing peptide-MHC class II interactions throughout the peptide-binding cleft. J. Biol. Chem..

[25] Yin L., Maben Z.J., Becerra A., Stern L.J. (2015). Evaluating the role of HLA-DM in MHC class II-peptide association reactions. J. Immunol..

[26] Lee H.K., Mattei L.M., Steinberg B.E., Alberts P., Lee Y.H., Chervonsky A., Mizushima N., Grinstein S., Iwasaki A. (2010). In vivo requirement for Atg5 in antigen presentation by dendritic cells. Immunity.

[27] Schmid D., Münz C. (2007). Innate and adaptive immunity through autophagy. Immunity.

[28] Paludan C., Schmid D., Landthaler M., Vockerodt M., Kube D., Tuschl T., Münz C. (2005). Endogenous MHC class II processing of a viral nuclear antigen after autophagy. Science.

[29] Kaushik S., Cuervo A.M. (2012). Chaperone-mediated autophagy: A unique way to enter the lysosome world. Trends Cell Biol..

[30] Gomes L.C., Dikic I. (2014). Autophagy in antimicrobial immunity. Mol. Cell.

[31] Klionsky D.J., Abdel-Aziz A.K., Abdelfatah S., Abdellatif M., Abdoli A., Abel S., Abeliovich H., Abildgaard M.H., Abudu Y.P., Acevedo-Arozena A. (2021). Guidelines for the use and interpretation of assays for monitoring autophagy. Autophagy.

[32] Vallejo A.N., Miller N.W., Clem L.W. (1991). Phylogeny of immune recognition: Role of alloantigens in antigen presentation in channel catfish immune responses. Immunology.

[33] Vallejo A.N., Miller N.W., Harvey N.E., Cuchens M.A., William Clem L. (1992). Cellular pathway(s) of antigen processing and presentation in fish APC: Endosomal involvement and cell-free antigen presentation. Dev. Immunol..

[34] Yamaguchi T., Dijkstra J.M. (2019). Major histocompatibility complex (MHC) genes and disease resistance in fish. Cells.

[35] Radwan J., Babik W., Kaufman J., Lenz T.L., Winternitz J. (2020). Advances in the evolutionary understanding of MHC polymorphism. Trends Genet..

[36] Kaufman J. (2018). Unfinished business: Evolution of the MHC and the adaptive immune system of jawed vertebrates. Annu. Rev. Immunol..

[37] Nonaka M., Naruse K., Matsuo M., Shima A. (2001). Comparative genomics of medaka: The major histocompatibility complex (MHC). Mar. Biotechnol..

[38] Kim D.-H., Lee S.-M., Hong B.-Y., Kim Y.-T., Choi T.-J. (2003). Cloning and sequence analysis of cDNA for the proteasome activator PA28-β subunit of flounder (*Paralichthys olivaceus*). Mol. Immunol..

[39] Liu G., Zheng W., Chen X. (2007). Molecular cloning of proteasome activator PA28-β subunit of large yellow croaker (*Pseudosciana crocea*) and its coordinated up-regulation with MHC class I α-chain and β2-microglobulin in poly I:C-treated fish. Mol. Immunol..

[40] Kasthuri S.R., Umasuthan N., Whang I., Kim E., Park H.-C., Lee J. (2013). Genomic structural characterization and transcriptional expression analysis of proteasome activator PA28α and PA28β subunits from Oplegnathus fasciatus. Fish Shellfish Immunol..

[41] Pinto R.D., Randelli E., Buonocore F., Pereira P.J.B., dos Santos N.M.S. (2013). Molecular cloning and characterization of sea bass (*Dicentrarchus labrax,* L.) MHC class I heavy chain and β2-microglobulin. Dev. Comp. Immunol..

[42] Pinto R.D., Moreira A.R., Pereira P.J.B., dos Santos N.M.S. (2013). Two thioredoxin-superfamily members from sea bass (*Dicentrarchus labrax*, L.): Characterization of PDI (PDIA1) and ERp57 (PDIA3). Fish Shellfish Immunol..

[43] Sever L., Bols N.C., Dixon B. (2013). The cloning and inducible expression of the rainbow trout ERp57 gene. Fish Shellfish Immunol..

[44] Sever L., Vo N.T.K., Bols N.C., Dixon B. (2014). Expression of tapasin in rainbow trout tissues and cell lines and up regulation in a monocyte/macrophage cell line (RTS11) by a viral mimic and viral infection. Dev. Comp. Immunol..

[45] Wilson W.H., Gilg I.C., Moniruzzaman M., Field E.K., Koren S., Lecleir G.R., Martínez-Martínez J., Poulton N.J., Swan B.K., Stepanauskas R. (2017). Genomic exploration of individual giant ocean viruses. ISME J..

[46] Nonaka M.I., Aizawa K., Mitani H., Bannai H.P., Nonaka M. (2011). Retained orthologous relationships of the MHC class I genes during euteleost evolution. Mol. Biol. Evol..

[47] Chen J., Wang L., Huang J., Li X., Guan L., Wang Q., Yang M., Qin Q. (2022). Functional analysis of a novel MHC-Iα genotype in orange-spotted grouper: Effects on Singapore grouper iridovirus (SGIV) replication and apoptosis. Fish Shellfish Immunol..

[48] Hashimoto K., Nakanishi T., Kurosawa Y. (1990). Isolation of carp genes encoding major histocompatibility complex antigens. Proc. Natl. Acad. Sci. USA.

[49] McConnell S.C., Hernández K.M., Wcisel D.J., Kettleborough R.N., Stemple D.L., Yoder J.A., Andrade J., de Jong J.L.O. (2016). Alternative haplotypes of antigen processing genes in zebrafish diverged early in vertebrate evolution. Proc. Natl. Acad. Sci. USA.

[50] Grimholt U. (2018). Whole genome duplications have provided teleosts with many roads to peptide loaded MHC class I molecules. BMC Evol. Biol..

[51] Dirscherl H., Yoder J.A. (2015). A nonclassical MHC class I U lineage locus in zebrafish with a null haplotypic variant. Immunogenetics.

[52] Grimholt U., Fosse J.H., Sundaram A.Y.M. (2020). Selective stimulation of duplicated Atlantic salmon MHC pathway genes by interferon-gamma. Front. Immunol..

[53] Cuesta A., Ángeles Esteban M., Meseguer J. (2006). Cloning, distribution and up-regulation of the teleost fish MHC class II alpha suggests a role for granulocytes as antigen-presenting cells. Mol. Immunol..

[54] Pilstrom L., Warr G.W., Stromberg S. (2005). Why is the antibody response of Atlantic cod so poor? The search for a genetic explanation. Fish. Sci..

[55] Haase D., Roth O., Kalbe M., Schmiedeskamp G., Scharsack J.P., Rosenstiel P., Reusch T.B.H. (2013). Absence of major histocompatibility complex class II mediated immunity in pipefish, *Syngnathus typhle*: Evidence from deep transcriptome sequencing. Biol. Lett..

[56] Dijkstra J.M., Yamaguchi T. (2019). Ancient features of the MHC class II presentation pathway, and a model for the possible origin of MHC molecules. Immunogenetics.

[57] Dijkstra J.M., Grimholt U., Leong J., Koop B.F., Hashimoto K. (2013). Comprehensive analysis of MHC class II genes in teleost fish genomes reveals dispensability of the peptide-loading DM system in a large part of vertebrates. BMC Evol. Biol..

[58] Fujiki K. (2003). Alternate forms of MHC class II-associated invariant chain are not produced by alternative splicing in rainbow trout (*Oncorhynchus mykiss*) but are encoded by separate genes. Dev. Comp. Immunol..

[59] Semple S.L., Heath G., Christie D., Braunstein M., Kales S.C., Dixon B. (2019). Immune stimulation of rainbow trout reveals divergent regulation of MH class II-associated invariant chain isoforms. Immunogenetics.

[60] Li M., Li Q., Yang Z., Hu G., Li T., Chen X., Ao J. (2014). Identification of cathepsin B from large yellow croaker (*Pseudosciaena crocea*) and its role in the processing of MHC class II-associated invariant chain. Dev. Comp. Immunol..

[61] Chen L., Zhang M., Sun L. (2011). Identification and expressional analysis of two cathepsins from half-smooth tongue sole (*Cynoglossus semilaevis*). Fish. Shellfish Immunol..

[62] Li Q., Ao J., Mu Y., Yang Z., Li T., Zhang X., Chen X. (2015). Cathepsin S, but not cathepsin L, participates in the MHC class II-associated invariant chain processing in large yellow croaker (*Larimichthys crocea*). Fish. Shellfish Immunol..

[63] Sun Y., Xu T., Wang J., Cheng Y., Wang R. (2011). Sequence and expression analysis of cathepsin S gene in the miiuy croaker *Miichthys miiuy*. Fish. Physiol. Biochem..

[64] Che R., Wang R., Xu T. (2014). Comparative genomic of the teleost cathepsin B and H and involvement in bacterial induced immunity of miiuy croaker. Fish. Shellfish Immunol..

[65] Dong X., Ye Z., Song L., Su B., Zhao H., Peatman E., Li C. (2016). Expression profile analysis of two cathepsin S in channel catfish (*Ictalurus punctatus*) mucosal tissues following bacterial challenge. Fish. Shellfish Immunol..

[66] Shen Y., Zhang H., Zhou Y., Sun Y., Yang H., Cao Z., Qin Q., Liu C., Guo W. (2021). Functional characterization of cathepsin B and its role in the antimicrobial immune responses in golden pompano (*Trachinotus ovatus*). Dev. Comp. Immunol..

[67] Criscitiello M.F., Ohta Y., Graham M.D., Eubanks J.O., Chen P.L., Flajnik M.F. (2012). Shark class II invariant chain reveals ancient conserved relationships with cathepsins and MHC class II. Dev. Comp. Immunol..

[68] Dijkstra J.M., Köllner B., Aoyagi K., Sawamoto Y., Kuroda A., Ototake M., Nakanishi T., Fischer U. (2003). The rainbow trout classical MHC class I molecule Onmy-UBA*501 is expressed in similar cell types as mammalian classical MHC class I molecules. Fish. Shellfish Immunol..

[69] Chang Y.T., Kai Y.H., Chi S.C., Song Y.L. (2011). Cytotoxic CD8α+ leucocytes have heterogeneous features in antigen recognition and class I MHC restriction in grouper. Fish. Shellfish Immunol..

[70] Kanako L. (2014). Lewis; Natasha, Del Cid; D.T. Perspectives on antigen presenting cells in zebrafish. Dev. Comp. Immunol..

[71] Iliev D.B., Thim H., Lagos L., Olsen R., Jørgensen J.B. (2013). Homing of antigen-presenting cells in head kidney and spleen—salmon head kidney hosts diverse APC types. Front. Immunol..

[72] Ronza P., Álvarez-Dios J.A., Robledo D., Losada A.P., Romero R., Bermúdez R., Pardo B.G., Martínez P., Quiroga M.I. (2021). Blood transcriptomics of turbot *Scophthalmus maximus*: A tool for health monitoring and disease studies. Animals.

[73] Pereiro P., Romero A., Díaz-Rosales P., Estepa A., Figueras A., Novoa B. (2017). Nucleated teleost erythrocytes play an Nk-lysin- and autophagy-dependent role in antiviral immunity. Front. Immunol..

[74] Lugo-Villarino G., Balla K.M., Stachura D.L., Bañuelos K., Werneck M.B.F., Traver D. (2010). Identification of dendritic antigen-presenting cells in the zebrafish. Proc. Natl. Acad. Sci. USA.

[75] Shao T., Zhu L.-Y., Nie L., Shi W., Dong W.-R., Xiang L.-X., Shao J.-Z. (2015). Characterization of surface phenotypic molecules of teleost dendritic cells. Dev. Comp. Immunol..

[76] Zoccola E., Delamare-Deboutteville J., Barnes A.C. (2015). Identification of barramundi (*Lates calcarifer*) DC-SCRIPT, a specific molecular marker for dendritic cells in fish. PLoS ONE.

[77] Soleto I., Fischer U., Tafalla C., Granja A.G. (2018). Identification of a potential common ancestor for mammalian cross-presenting dendritic cells in teleost respiratory surfaces. Front. Immunol..

[78] Soleto I., Granja A.G., Simón R., Morel E., Díaz-Rosales P., Tafalla C. (2019). Identification of CD8α+ dendritic cells in rainbow trout (*Oncorhynchus mykiss*) intestine. Fish. Shellfish Immunol..

[79] Kato G., Miyazawa H., Nakayama Y., Ikari Y., Kondo H., Yamaguchi T., Sano M., Fischer U. (2018). A novel antigen-sampling cell in the teleost gill epithelium with the potential for direct antigen presentation in mucosal tissue. Front. Immunol..

[80] Hu Y., Li A., Xu Y., Jiang B., Lu G., Luo X. (2017). Transcriptomic variation of locally-infected skin of Epinephelus coioides reveals the mucosal immune mechanism against *Cryptocaryon irritans*. Fish. Shellfish Immunol..

[81] Wang H., Chen X., Li S., Zhou C., Xu L., Wu Z., Chen X. (2021). Identification and expression analysis of Langerhans cells marker Langerin/CD207 in grass carp, *Ctenopharyngodon idella*. Gene.

[82] Wu L., Qin Z., Liu H., Lin L., Ye J., Li J. (2020). Recent advances on phagocytic B cells in teleost fish. Front. Immunol..

[83] Miller N., Wilson M., Bfuflten E., Stuge T., Warr G., Ciem W. (1998). Functional and molecular characterization of teleost leukocytes. Immunol. Rev..

[84] Abós B., Castro R., González Granja A., Havixbeck J.J., Barreda D.R., Tafalla C. (2015). Early activation of teleost B cells in response to rhabdovirus infection. J. Virol..

[85] Castro R., Abós B., González L., Granja A.G., Tafalla C. (2017). Expansion and differentiation of IgM+ B cells in the rainbow trout peritoneal cavity in response to different antigens. Dev. Comp. Immunol..

[86] Sunyer J.O. (2012). Evolutionary and functional relationships of B cells from fish and mammals: Insights into their novel roles in phagocytosis and presentation of particulate antigen. Infect. Disord. Drug Targets.

[87] Rougeot J., Torraca V., Zakrzewska A., Kanwal Z., Jansen H.J., Sommer F., Spaink H.P., Meijer A.H. (2019). RNAseq profiling of leukocyte populations in zebrafish larvae reveals a cxcl11 chemokine gene as a marker of macrophage polarization during mycobacterial infection. Front. Immunol..

[88] Sever L., Vo N.T.K., Bols N.C., Dixon B. (2018). Tapasin’s protein interactions in the rainbow trout peptide-loading complex. Dev. Comp. Immunol..

[89] Cui X., Ji C., Cao X., Fu Z., Zhang S., Guo X. (2012). Molecular and biological characterization of interferon-γ-inducible-lysosomal thiol reductase gene in zebrafish (Danio rerio). Fish. Shellfish Immunol..

[90] Zheng W., Chen X. (2006). Cloning and expression analysis of interferon-γ-inducible-lysosomal thiol reductase gene in large yellow croaker (*Pseudosciaena crocea*). Mol. Immunol..

[91] Song J., Liu H., Ma L., Gao C., Zhang S. (2014). Molecular cloning, expression and functional characterization of interferon-γ-inducible lysosomal thiol reductase (GILT) gene from mandarin fish (*Siniperca chuatsi*). Fish. Shellfish Immunol..

[92] Herpin A., Lescat L., Bobe J., Jenny A., Seiliez I. (2020). Lighting chaperone-mediated autophagy (CMA) evolution with an ancient LAMP: The existence of a functional CMA activity in fish. Autophagy.

[93] Lescat L., Véeron V., Mourot B., Péron S., Chenais N., Dias K., Riera-Heredia N., Beaumatin F., Pinel K., Priault M. (2020). Chaperone-mediated autophagy in the light of evolution: Insight from fish. Mol. Biol. Evol..

[94] Lescat L., Herpin A., Mourot B., Véron V., Guiguen Y., Bobe J., Seiliez I. (2018). CMA restricted to mammals and birds: Myth or reality?. Autophagy.

[95] Yabu T., Imamura S., Mohammed M.S., Touhata K., Minami T., Terayama M., Yamashita M. (2011). Differential gene expression of HSC70/HSP70 in yellowtail cells in response to chaperone-mediated autophagy. FEBS J..

[96] Dubińska-Magiera M., Niedbalska-Tarnowska J., Migocka-Patrzałek M., Posyniak E., Daczewska M. (2020). Characterization of Hspb8 in Zebrafish. Cells.

[97] Pant D.C., Nazarko T.Y. (2021). Selective autophagy: The rise of the zebrafish model. Autophagy.

[98] Muñoz-Sánchez S., van der Vaart M., Meijer A.H. (2020). Autophagy and Lc3-associated phagocytosis in zebrafish models of bacterial infections. Cells.

[99] Valionyte E., Yang Y., Griffiths S.A., Bone A.T., Barrow E.R., Sharma V., Lu B., Luo S. (2021). The caspase-6–p62 axis modulates p62 droplets based autophagy in a dominant-negative manner. Cell Death Differ..

[100] Dong G., Zhang Z., Duan K., Shi W., Huang R., Wang B., Luo L., Zhang Y., Ruan H., Huang H. (2020). Beclin 1 deficiency causes hepatic cell apoptosis via endoplasmic reticulum stress in zebrafish larvae. FEBS Lett..

[101] Mawed S.A., He Y., Zhang J., Mei J. (2020). Strategy of hepatic metabolic defects induced by beclin1 heterozygosity in adult zebrafish. Int. J. Mol. Sci..

[102] Masud S., Prajsnar T.K., Torraca V., Lamers G.E.M., Benning M., Van Der Vaart M., Meijer A.H. (2019). Macrophages target Salmonella by Lc3-associated phagocytosis in a systemic infection model. Autophagy.

[103] Prajsnar T.K., Serba J.J., Dekker B.M., Gibson J.F., Masud S., Fleming A., Johnston S.A., Renshaw S.A., Meijer A.H. (2021). The autophagic response to *Staphylococcus aureus* provides an intracellular niche in neutrophils. Autophagy.

[104] Gibson J.F., Prajsnar T.K., Hill C.J., Tooke A.K., Serba J.J., Tonge R.D., Foster S.J., Grierson A.J., Ingham P.W., Renshaw S.A. (2021). Neutrophils use selective autophagy receptor Sqstm1/p62 to target *Staphylococcus aureus* for degradation in vivo in zebrafish. Autophagy.

[105] Lu X., Li W., Guo J., Jia P., Zhang W., Yi M., Jia K. (2022). N Protein of viral hemorrhagic septicemia virus suppresses STAT1-mediated MHC class II transcription to impair antigen presentation in sea perch, *Lateolabrax japonicus*. J. Immunol..

[106] Abram Q.H., Vo N.T.K., Kellendonk C., Bols N.C., Katzenback B.A., Dixon B. (2020). Regulation of endogenous antigen presentation in response to suboptimal temperatures in a walleye skin fibroblast cell line. Fish Shellfish Immunol..

[107] Abram Q.H., Rodríguez-Ramos T., Bols N.C., Katzenback B.A., Dixon B. (2019). Effect of suboptimal temperature on the regulation of endogenous antigen presentation in a rainbow trout hypodermal fibroblast cell line. Dev. Comp. Immunol..

[108] Nombela I., Requena-platek R., Morales-lange B., Chico V., Puente-Marín S., Ciordia S., Mena M.C., Coll J., Perez L., Mercado L. (2019). Rainbow trout red blood cells exposed to viral hemorrhagic septicemia virus up-regulate antigen-processing mechanisms and MHC I&II, CD86, and CD83 antigen-presenting cell markers. Cells.

[109] Nombela I., López-Lorigados M., Salvador-Mira M.E., Puente-Marin S., Chico V., Ciordia S., Mena M.C., Mercado L., Coll J., Perez L. (2019). Integrated transcriptomic and proteomic analysis of red blood cells from rainbow trout challenged with VHSV point towards novel immunomodulant targets. Vaccines.

[110] Kong H.J., Moon J.-Y., Nam B.-H., Kim Y.-O., Kim W.-J., Lee J.-H., Kim K.-K., Kim B.-S., Yeo S.-Y., Lee C.H. (2011). Molecular characterization of the autophagy-related gene Beclin-1 from the olive flounder (*Paralichthys olivaceus*). Fish Shellfish Immunol..

[111] Peruzza L., Pascoli F., Dalla Rovere G., Franch R., Ferraresso S., Babbucci M., Biasini L., Abbadi M., Panzarin V., Toffan A. (2021). Transcriptome analysis reveals a complex response to the RGNNV/SJNNV reassortant Nervous Necrosis Virus strain in sea bream larvae. Fish Shellfish Immunol..

[112] Rao Y., Wan Q., Su H., Xiao X., Liao Z., Ji J., Yang C., Lin L., Su J. (2018). ROS-induced HSP70 promotes cytoplasmic translocation of high-mobility group box 1b and stimulates antiviral autophagy in grass carp kidney cells. J. Biol. Chem..

[113] Hansen J., La Patra S. (2002). Induction of the rainbow trout MHC class I pathway during acute IHNV infection. Immunogenetics.

[114] Svenning S., Gondek-Wyrozemska A.T., van der Wal Y.A., Robertsen B., Jensen I., Jørgensen J.B., Edholm E.-S. (2019). Microbial danger signals control transcriptional induction of distinct MHC class I L lineage genes in Atlantic salmon. Front. Immunol..

[115] Wang B., Du H., Huang H., Xian J., Xia Z., Hu Y. (2019). Major histocompatibility complex class I (MHC Iα) of Japanese flounder (*Paralichthys olivaceus*) plays a critical role in defense against intracellular pathogen infection. Fish Shellfish Immunol..

[116] Ahn D.-H., Kang S., Park H. (2016). Transcriptome analysis of immune response genes induced by pathogen agonists in the Antarctic bullhead notothen *Notothenia coriiceps*. Fish Shellfish Immunol..

[117] Bello-Pérez M., Pereiro P., Coll J., Novoa B., Pérez L., Falco A. (2020). Zebrafish C-reactive protein isoforms inhibit SVCV replication by blocking autophagy through interactions with cell membrane cholesterol. Sci. Rep..

[118] Pereiro P., Figueras A., Novoa B. (2020). Zebrafish pten genes play relevant but distinct roles in antiviral immunity. Vaccines.

[119] Peatman E., Terhune J., Baoprasertkul P., Xu P., Nandi S., Wang S., Somridhivej B., Kucuktas H., Li P., Dunham R. (2008). Microarray analysis of gene expression in the blue catfish liver reveals early activation of the MHC class I pathway after infection with *Edwardsiella ictaluri*. Mol. Immunol..

[120] Li K., Wei X., Zhang L., Chi H., Yang J. (2019). Raptor/mTORC1 acts as a modulatory center to regulate anti-bacterial immune response in rockfish. Front. Immunol..

[121] Kumar G., Hummel K., Noebauer K., Welch T.J., Razzazi-Fazeli E., El-Matbouli M. (2018). Proteome analysis reveals a role of rainbow trout lymphoid organs during Yersinia ruckeri infection process. Sci. Rep..

[122] Lü A., Hu X., Xue J., Zhu J., Wang Y., Zhou G. (2012). Gene expression profiling in the skin of zebrafish infected with Citrobacter freundii. Fish Shellfish Immunol..

[123] Wu X.M., Cao L., Hu Y.W., Chang M.X. (2019). Transcriptomic characterization of adult zebrafish infected with Streptococcus agalactiae. Fish Shellfish Immunol..

[124] Li Y., Wang T., Gao S., Xu G., Niu H., Huang R., Wu S. (2016). Salmonella plasmid virulence gene spvB enhances bacterial virulence by inhibiting autophagy in a zebrafish infection model. Fish Shellfish Immunol..

[125] Young N.D., Cooper G.A., Nowak B.F., Koop B.F., Morrison R.N. (2008). Coordinated down-regulation of the antigen processing machinery in the gills of amoebic gill disease-affected Atlantic salmon (*Salmo salar* L.). Mol. Immunol..

[126] Tadiso T.M., Krasnov A., Skugor S., Afanasyev S., Hordvik I., Nilsen F. (2011). Gene expression analyses of immune responses in Atlantic salmon during early stages of infection by salmon louse (*Lepeophtheirus salmonis*) revealed bi-phasic responses coinciding with the copepod-chalimus transition. BMC Genom..

[127] Konczal M., Ellison A.R., Phillips K.P., Radwan J., Mohammed R.S., Cable J., Chadzinska M. (2020). RNA-Seq analysis of the guppy immune response against *Gyrodactylus bullatarudis* infection. Parasite Immunol..

[128] Cervera L., González-Fernández C., Arizcun M., Cuesta A., Chaves-Pozo E. (2022). Severe natural outbreak of *Cryptocaryon irritans* in gilthead seabream produces leukocyte mobilization and innate immunity at the gill tissue. Int. J. Mol. Sci..

[129] Sun Z., Shen B., Wu H., Zhou X., Wang Q., Xiao J., Zhang Y. (2015). The secreted fructose 1,6-bisphosphate aldolase as a broad spectrum vaccine candidate against pathogenic bacteria in aquaculture. Fish Shellfish Immunol..

[130] Darawiroj D., Kondo H., Hirono I., Aoki T. (2008). Immune-related gene expression profiling of yellowtail (*Seriola quinqueradiata*) kidney cells stimulated with ConA and LPS using microarray analysis. Fish Shellfish Immunol..

[131] Mostowy S., Boucontet L., Mazón-Moya M.J., Sirianni A., Boudinot P., Hollinshead M., Cossart P., Herbomel P., Levraud J.P., Colucci-Guyon E. (2013). The zebrafish as a new model for the in vivo study of *Shigella flexneri* interaction with phagocytes and bacterial autophagy. PLoS Pathog..

[132] Meijer A.H., van der Vaart M. (2014). DRAM1 promotes the targeting of mycobacteria to selective autophagy. Autophagy.

[133] Van der Vaart M., Korbee C.J., Lamers G.E.M., Tengeler A.C., Hosseini R., Haks M.C., Ottenhoff T.H.M., Spaink H.P., Meijer A.H. (2014). The DNA damage-regulated autophagy modulator DRAM1 links mycobacterial recognition via TLP-MYD88 to authophagic defense. Cell Host Microbe.

[134] Stockhammer O.W., Rauwerda H., Wittink F.R., Breit T.M., Meijer A.H., Spaink H.P. (2010). Transcriptome analysis of Traf6 function in the innate immune response of zebrafish embryos. Mol. Immunol..

[135] Yang D., Liu Q., Yang M., Wu H., Wang Q., Xiao J., Zhang Y. (2012). RNA-seq liver transcriptome analysis reveals an activated MHC-I pathway and an inhibited MHC-II pathway at the early stage of vaccine immunization in zebrafish. BMC Genom..

[136] Yang D., Liu Q., Ni C., Li S., Wu H., Wang Q., Xiao J., Zhang Y. (2013). Gene expression profiling in live attenuated *Edwardsiella tarda* vaccine immunized and challenged zebrafish: Insights into the basic mechanisms of protection seen in immunized fish. Dev. Comp. Immunol..

[137] Gao Y., Wu H., Wang Q., Qu J., Liu Q., Xiao J., Zhang Y. (2014). A live attenuated combination vaccine evokes effective immune-mediated protection against *Edwardsiella tarda* and *Vibrio anguillarum*. Vaccine.

[138] Zhang X., Mu Y., Mu P., Ao J., Chen X. (2017). Transcriptome analysis reveals comprehensive insights into the early immune response of large yellow croaker (*Larimichthys crocea*) induced by trivalent bacterial vaccine. PLoS ONE.

[139] García-Valtanen P., Del Mar Ortega-Villaizan M., Martínez-López A., Medina-Gali R., Pérez L., Mackenzie S., Figueras A., Coll J.M., Estepa A. (2014). Autophagy-inducing peptides from mammalian VSV and fish VHSV rhabdoviral G glycoproteins (G) as models for the development of new therapeutic molecules. Autophagy.

[140] Espín-Palazón R., Martínez-López A., Roca F.J., López-Muñoz A., Tyrkalska S.D., Candel S., García-Moreno D., Falco A., Meseguer J., Estepa A. (2016). TNFα impairs rhabdoviral clearance by inhibiting the host autophagic antiviral response. PLoS Pathog..

